# Overlapping Infection by *Strongyloides* spp. and Cytomegalovirus in the Immunocompromised Host: A Comprehensive Review of the Literature

**DOI:** 10.3390/tropicalmed8070358

**Published:** 2023-07-10

**Authors:** Tommaso Lupia, Elena Crisà, Alberto Gaviraghi, Barbara Rizzello, Alessia Di Vincenzo, Fabrizio Carnevale-Schianca, Daniela Caravelli, Marco Fizzotti, Francesco Tolomeo, Umberto Vitolo, Ilaria De Benedetto, Nour Shbaklo, Alessandro Cerutti, Piero Fenu, Vanesa Gregorc, Silvia Corcione, Valeria Ghisetti, Francesco Giuseppe De Rosa

**Affiliations:** 1 Unit of Infectious Diseases, Cardinal Massaia, 14100 Asti, Italy; 2 Unit of Oncology and Haematology, Candiolo Cancer Institute (FPO-IRCCS), Strada Provinciale 142, Km 3,95, 10060 Candiolo, Italy; 3 Department of Medical Sciences, Infectious Diseases, University of Turin, 10126 Turin, Italy; 4 Microbiology Unit, Amedeo di Savoia Hospital, 10100 Turin, Italy; 5 Intensive Care Unit, IRCCS Candiolo, 10100 Candiolo, Italy; 6 Healthcare Management, IRCCS Candiolo, 10100 Candiolo, Italy; 7 School of Medicine, Tufts University, Boston, MA 02111, USA

**Keywords:** *Strongyloides*, cytomegalovirus, immunocompromised host, solid organ transplant, hematopoietic stem-cell transplantation, HIV

## Abstract

*Strongyloides* and cytomegalovirus co-infections are rarely reported, even though they are distinguished by high morbidity and mortality, especially in immunocompromised hosts. We narratively reviewed the literature on reported cases of *Strongyloides* and CMV co-infections in immunosuppressed patients. Most cases occurred in males with a median age of 47 (IQR, 37–59). *Strongyloides*/CMV co-infections occurred among immunocompromised hosts, especially in solid organ transplants and hematological or rheumatological diseases. Most of the patients underwent a course of steroid treatment before the diagnosis of co-infections. Other common immunomodulatory agents were tacrolimus and mycophenolate. The first clinical manifestations of co-infections were mainly gastrointestinal, followed by respiratory symptoms. CMV was, in most patients, co-infected with an isolated reactivation, although *Strongyloides* manifested especially as hyperinfection syndrome. Ganciclovir and ivermectin are the mainstays of CMV and *Strongyloides* treatment. However, the treatment mortality reported in this narrative review is around 52.4%. Interestingly secondary bacterial infections are common in CMV/*Strongyloides*-infected patients.

## 1. Introduction

Strongyloidiasis is a parasitic infection caused by the nematode *Strongyloides stercoralis* (and less commonly by *Strongyloides fülleborni*) [1]. It is widely endemic in tropical and subtropical areas but is also present in temperate areas. According to the WHO, the number of infections worldwide is 30–100.000 million, which is probably underestimated [2,3]. The most common method of transmission is contact with larvae of *Strongyloides*, which live in soil and can penetrate into the skin [1,2]. Then, through bloodstream and lymphatic circulation, they migrate into lung tissues and ascend the airways until they are swallowed and reach the gut, where they mature into adult forms. Adult worms deposit eggs in intestinal mucosa, which become larvae that can be excreted in stools and cause an autoinfection cycle through reinvading intestinal walls, potentially carrying intestinal bacteria into the bloodstream or perianal skin [1,2].

Disease burden is different according to the immune status of patients [4,5]. Clinical manifestations in immunocompetent hosts are usually mild and chronic, such as gastrointestinal (pain, diarrhea, vomiting), cutaneous (larva currens, orticaria, rash) and respiratory symptoms (cough, dyspnea). Severe manifestations occur in immunocompromised patients and include hyperinfection syndrome (HS) and disseminated strongyloidiasis (DS) [4,5]. HS consists of a massive replication of parasites within the host, which can lead to intestinal obstruction, peritonitis, gastrointestinal bleeding, pneumonitis, alveolar hemorrhage, respiratory failure, or sepsis. DS happens when parasites invade tissues outside the usual sites of replication [4,5]. Undergoing high-dose corticosteroid therapies or immunosuppressive regimens after solid organ transplant, hematopoietic stem-cell transplant (HSCT), and other immune deficits (HIV, HTLV-1) are risk factors for developing HS and DS, related to a high mortality rate (up to 80–90%) [4,5].

Immunocompromised individuals are also at risk of co-infection with other opportunistic pathogens, including Cytomegalovirus (CMV). CMV is a widely spread virus, which about 60–70% of adults in industrialized countries and close to 100% in emerging countries experience lifelong exposure. Transmission occurs through organ transplantation of an infected organ or contact with infected bodily secretions [6]. The spectrum of its clinical manifestation is wide: primary infection often runs asymptomatic or paucisymptomatic in an immunocompetent host [7]. When symptomatic, the most common presentation is a mononucleosis-like syndrome (i.e., fever, asthenia, rash, lymphadenopathy) and single-organ involvement is unusual. Similar to other members of Herpesviridae, CMV stays latent in myeloid and endothelial cells. As such, immunosuppression settings, mainly those resulting in altered T-lymphocyte response, can lead to a greater risk of reactivation; specific organ diseases include colitis, esophagitis, hepatitis, pneumonia, encephalitis, retinitis, adrenalitis, and pericarditis/myocarditis as a consequence of broad virus cellular tropism. Disseminated disease represents the most severe clinical entity, carrying significantly poor outcomes [6,7,8].

We carried out a narrative review aimed at examining previously reported cases of *Strongyloides* spp. and CMV concomitant infections in immunosuppressed hosts.

## 2. Materials and Methods

**Figure 1:**
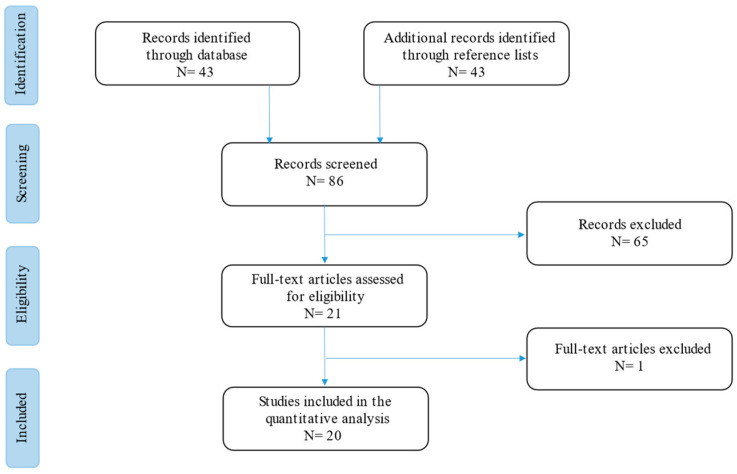
Flow-chart of the studies revised in the narrative review.

The current narrative review followed the Scale for the Assessment of Narrative Review Articles (SANRA) flow-chart (Figure 1) [9].

The main aim of this work was to summarize current evidence on *Strongyloides* and CMV co-infections in immunosuppressed patients to understand clinical characteristics, treatment, and outcome.

A search was run on Cochrane, PubMed, and Google Scholar using the terms (‘*Strongyloides*’ [Mesh]) AND (‘Cytomegalovirus’ [Mesh]) OR (‘CMV’ [Mesh]), (‘*Strongyloides*’ [Mesh]) AND (‘Transplant’ [Mesh]), (‘*Strongyloides*’ [Mesh]) AND (‘Solid Organ Transplant’ [Mesh]), (‘*Strongyloides*’ [Mesh]) AND (‘HSCT’ [Mesh]) and (‘*Strongyloides*’ [Mesh]) AND (‘HIV’ [Mesh]) in English. Results were limited to those published between 1 January 1980 and 1 January 2023. Studies were filtered for practice guidelines, guidelines, meta-analyses, systematic reviews, narrative reviews, case series, and case reports (Figure 1).

Our search strategy permitted the identification of 43 papers, of which 23 were excluded by title and abstract evaluation. Full-text papers were then assessed for eligibility according to the above criteria, and the results are included in Table 1.

Then, the reviewers studied titles and abstracts. Subsequently, 86 papers were included. Finally, quality assessment of full-text studies was performed by two independent reviewers (AG and TL). Researchers reviewed the summary of all articles sought and ultimately used data from 20 full articles to compile this review paper. Researchers assessed the inclusion of all titles and abstracts without language limitations in English. We duplicated other studies previously included and excluded papers with no methods described, along with papers not strictly related to the aim of the study and according to journal importance and the number of references.

We performed descriptive statistics on the entire study population. Data were analyzed using standard statistical methods. Variables were described with medians, absolute values, and rates.

## 3. Results

The 20 manuscripts included were all in the editorial form of case reports [10,11,12,13,14,15,16,17,18,19,20,21,22,23,24,25,26,27,28,29,30], barring the retrospective analysis by Miglioli-Galvão and colleagues [30], and 31 patients were included. The paper by Miglioli-Galvão et al. did not include clinical and microbiological data of the patients (n = 10), and for this reason it was excluded from the final analysis presented in Table 2.

Most of the patients were from the U.S. (n = 6; 28.57%) or India (four patients; 19.04%), although in two cases, the origin of the patients was lacking and was defined only as Caucasian.

The median age at diagnosis in this population was 47 (IQR 37–59; range 20–68) years old and there was a higher prevalence of males (n = 14; 77.77%).

The primary underlying diagnosis in this immunosuppressed population was solid organ transplantation (n = 12; 57%): nine were kidney transplants, which accounted for 75% of all the transplants, and there was one liver, one heart, and one pancreas transplant. The second underlying diagnosis was hematological diseases (n = 3), as presented in Table 1. It is worth noting that one patient did not have any classic predisposing immunodeficiency cause, but he was exposed to steroid therapy.

Data regarding immunosuppressant therapies are lacking from a large part of the population described. However, 15 patients (71.24%) were reported to be treated with a long course of steroids, and the most frequent non-steroid immunosuppressors prescribed were tacrolimus (n = 9; 42.8%) and MMF (n = 8; 38.1%).

Comorbidities other than the cause of immunosuppression were reported in six cases only and were mainly hypertension (n = 4) and diabetes mellitus (n = 2).

Signs and symptoms at presentation were divided into three main groups: gastrointestinal, skin, and respiratory. Gastrointestinal symptoms were reported in sixteen patients (76.2%), skin manifestations in three (14.2%), and respiratory symptoms in six patients (28.57%).

In most of the patients (76.1%), isolated CMV viral reactivation was identified, and only five patients (23.8%) had organ (or multiple) localization.

Hyperinfection syndrome by *S. stercoralis* was the most common clinical manifestation of the strongyloidiasis disease, being reported in 14 patients (66.7%), followed by only gastrointestinal localization in 6 patients (28.5%).

Eosinophils count was rarely reported (i.e., in three reports) and resulted within the normal limits.

Almost half (12; 57.1%) of the patients received treatment for both CMV and *S. stercoralis*. In fact, nine patients (42.8%) had not been treated for CMV reactivation, and ganciclovir was used as the first-line antiviral drug in only ten patients (47.6%), whereas valganciclovir was used in two (9.5%).

The most-used anthelminthic drug was Ivermectin, which was used in 14 patients (66.7%), of which 11 used it alone and 3 (14.2%) used it in combination with other antiparasitic drugs; albendazole or thiabendazole was used as single-drug therapy in 3 patients (14.2%, 9 both).

HS has been reported to be associated with Gram-negative bloodstream infections with a knight–horse mechanism. However, in the casuistry collected, a concomitant bacterial infection was reported in only nine patients (42.8%), and five of these (23.8%) were caused by Gram-negative bacteria.

More than half of the patients (11; 52.4%) with HS and CMV infection had a poor outcome, in line with what has been described in the literature for single *Strongyloides* hyperinfection.

## 4. Discussion

Concomitant infection with *Strongyloides* spp. and CMV is rare and has been reported in a few immunosuppressed patients in over 43 years of the reviewed literature. In this narrative review, we aimed to collect and discuss previously reported cases of *Strongyloides* spp. and CMV co-infections in immunosuppressed hosts.

CMV infection and reactivation risk are homogeneously present worldwide, although *Strongyloides* epidemiology varies according to country and state. In our review, most of the cases reported occurred in American (North or South) or Asian countries. According to Buonfrate and colleagues [2] in their systematic review of strongyloidiasis prevalence, Southeast Asian regions and American countries ranked first and third areas for the prevalence of *S. stercoralis* infection, with 6.9% (95% CI: 3.5–10.2%) and 12.1% (6.1–17.9%), respectively. Moreover, European countries presented a lower prevalence according to the same systematic review, with a rate of 2.8% (1.4–4.1%) [2].

In addition, according to the European Center for Diseases Prevention and Control (ECDC), in Italy, Spain, and France, the prevalence of rates of *S. stercoralis* infections was stated to be higher than the data reported by Buonfrate et al. and is estimated to be between 3.3% and 5.6% [10].

Most of the cases presented in this review occurred in males [10,11,12,13,14,15,16,17,18,19,20,21,22,23,24,25,26,27,28,29,30]. Females, in fact, comprised one third of the total cases reviewed, and the median age in women was 59 (46–66) years [10,11,12,13,14,15,16,17,18,19,20,21,22,23,24,25,26,27,28,29,30]. Interestingly, Arakaki and colleagues [31] compared high-endemic groups and lower-endemic groups in Okinawa (Japan), showing that patients were older at the time of *Strongyloides* diagnosis in a setting with lower prevalence.

We report a higher incidence of *Strongyloides*/CMV co-infection in renal transplants [10,11,12,13,14,15,16,17,18,19,20,21,22,23,24,25,26,27,28,29,30]. It is not possible to determine the precise incidence of infection caused by *Strongyloides* after solid organ transplantation. While infections have been documented in recipients of liver, heart, lung, heart–kidney, intestine, and pancreas transplants, the incidence of infections following kidney transplantation has been significantly higher than that of other organ transplants. This may be the result of a number of factors, including the frequency with which kidney transplantation is performed, the discovery of *S. stercoralis* organisms in the urine, or the intraperitoneal positioning of the transplanted kidney, which makes it especially vulnerable to invasion by extraintestinal *Strongyloides* [4,32].

Furthermore, CMV is among the most significant pathogens after SOT and HSCT. Renal transplantation and lung transplants, despite routine prophylaxis against CMV, are characterized by a high risk of reactivation, especially in seronegative recipients of seropositive donors (D+/R−) and seropositive recipients (R+) [33].

In our search, we found only one patient with a diagnosis of lymphoma and a concomitant *Strongyloides* and CMV co-infection [10]. Nonetheless, in the literature, different groups have summarized evidence of *Strongyloides* infection alone in lymphoma patients. Genta and colleagues [34] collected 17 cases of *Strongyloides* infection in lymphoma patients, most of them (n = 11) occurring after chemotherapy with (n = 9) or without (n = 2) steroids in treatment regimens. In subsequent work, Aydin and colleagues [35] reported a large part of cases shared with the previous work by Genta et al. [34] Interestingly, of the twenty patients collected, only one patient was in a European country (i.e., France).

Nucci et al. [36], in a retrospective study that involved 253 patients with hematological malignancy (mostly lymphoma), reported *Strongyloides* hyperinfection in 53 (21.0%) patients and fatal disseminated syndrome in 1 patient (1.9%). More recently, Abdelrahman and colleagues [37] reported a case of strongyloidiasis in angioimmunoblastic T-cell lymphoma with a review of the literature on cases occurring in recent years; five new cases were reported between 2000 and 2012.

Immunosuppressive conditions other than onco-hematological diseases such as HIV were reported in only two patients co-infected with *Strongyloides* and CMV. Schar and colleagues [38] highlighted how people living with HIV have twice the risk of *S. stercoralis* infection compared to the HIV-negative population.

Most of the patients collected in this systematic review underwent immunosuppressant regimens including steroids [10,11,12,13,14,15,16,17,18,19,20,21,22,23,24,25,26,27,28,29,30]. It has commonly been reported that there is a connection between corticosteroid treatment and strongyloidiasis [38,39,40]. Although corticosteroid treatment is linked to a two- to three-fold increase in the chance of being infected by *S. stercoralis*, the immunosuppression induced by corticosteroids is a condition that causes severe forms of the disease to manifest in patients who were previously asymptomatic [38,39,40]. Corticosteroids, due to their suppressive effects on some of the primary mediators of the immune response to *S. stercoralis* larvae, such as eosinophils, have been hypothesized to increase a host’s susceptibility to parasitic infection [41].

Interestingly, the risk of *Strongyloides* reactivation is also still high in patients receiving a short course of steroids, as reported in the literature.

Moreover, CMV infection causes transient but substantial immunosuppression [42,43]. CMV causes immunosuppression in recipients of solid organ transplants, which facilitates superinfections with various pathogens [42,43]. Notably, meta-analyses of thousands of transplant recipients have shown that anti-CMV prophylaxis prevents bacterial and fungal infections, as well as bacterial and protozoan infections [43,44].

In this review, we also found a large number of patients undergoing a course of tacrolimus and/or MMF before CMV and HS. Nolan and colleagues [45] found that instead of other widely used immunosuppressants such as cyclosporine, tacrolimus does not have anthelmintic activity against *S. stercoralis*, increasing the risk of reactivation in vulnerable patients. In our review, no cases of cyclosporine regimens with CMV/HS co-infections were reported, and the protective effect was also confirmed in larger studies.

Only a few cases reported patients’ comorbidities other than the immunosuppressive underlying conditions in this review; hypertension, DM, and hypothyroidism were the only ones recorded [46,47,48]. Several risk factors for *Strongyloides* infection are reported in the literature; however, there is a lack of studies focusing on specific demographic groups [46,47,48]. Geo-climatic and socio-economic factors, poor sanitation, adult age, and male sex are the most reported risk factors for *S. stercoralis* infection. However, other underlying conditions other than classical immunosuppression have been associated with higher risk of infection, such as diabetes, hypochlorhydria, alcoholism, tuberculosis, malnourishment, COPD, and renal failure [49,50,51,52]. If the association between *Strongyloides* and hypertension or hypothyroidism has only been described in single case reports, the role of diabetes mellitus in *Strongyloides* infection is still debated. An English retrospective case-control study reported a significantly higher prevalence of *Strongyloides* seropositivity in migrant patients with diabetes compared to those without it [50]. However, in several other studies in Thailand, Australia, and India, type 2 DM exhibited an inverse correlation with *S. stercoralis* infection [49,50,51]. This association could be explained by the immune response to the parasite infection. Helminth infections could be responsible for the alteration of the Th1/Th2 balance, leading to a reduction in the circulating Th17, reduced proinflammatory cytokine, and increased M2 macrophages; all of these immunologic mechanisms, associated with an altered gut microbiome, could lead to increased insulin sensitivity [52,53,54].

The revision of the literature highlighted that In patients with overlapping *Strongyloides*/CMV infections, the most reported symptoms were gastrointestinal, followed by respiratory symptoms [10,11,12,13,14,15,16,17,18,19,20,21,22,23,24,25,26,27,28,29,30]. Clinical presentations of *Strongyloides* and CMV disseminated infections are troublesome due to the overlaps of many symptoms commonly presented in both diseases. Epidemiology, lack of response to empirical CMV treatment, and rapid worsening of symptoms should raise suspicions of an overlapping syndrome in an immunosuppressed host, especially during or after recent corticosteroid treatment.

Interestingly in the cases analyzed for this review, eosinophil count was rarely reported and in most of cases proved to be normal. In an immunocompromised host, especially in a hematological patient, in patients previously or chronically treated with steroids, and in the case of disseminated disease, the risk of a normal eosinophils count is very high [55,56]. Buonfrate et al. in their systematic review of severe cases stated that eosinophilia was present in the 22.5% of the total cases of *Strongyloides* infections and only in 16.4% of disseminated diseases [57].

Interestingly, we found 76.1% of patients in which CMV viremia was detected without proven organ involvement. As described before, CMV reactivation facilitates superinfections with various pathogens, including parasites such as *Strongyloides*. Moreover, among the seven patients with concomitant *Strongyloides* infection and CMV disseminated disease as reported in our patient, the mortality was 85.74% versus 35.71% in patients with concomitant HS/DS and viral CMV isolated reactivation or the sole gastrointestinal disease. We hypothesize that CMV disseminated disease increases the mortality of patients with HS/DS disease, which is itself a disease with intrinsic high morbidity and mortality risk. In fact, HS and disseminated strongyloidiasis in immunosuppressed patients are life-threatening conditions, usually associated with a poor outcome, even with early recognition and treatment. As reported in the literature, mortality has declined in the last decade, with rates that range between 28.3 and 68.5% [58]. Solid organ transplantation and disseminated strongyloidiasis have been observed to be related to a higher mortality rate compared to HSCT and HS, even though not all the studies reach a statistical difference [4,5].

Almost half of the patients received treatment for both the CMV and *S. stercoralis*, and this could be related to at least three diagnoses post-mortem of overlapping syndromes and many isolated CMV reactivations that were not considered to worth antiviral treatment.

In 2016, the Cochrane Library and Henriquez-Camacho et al. [59] conducted a systematic collection of data regarding the treatment of strongyloidiasis with ivermectin and either albendazole or thiabendazole. The authors included 7 studies with a total of 1147 individuals hailing from a variety of nations [59]. In comparison to the data on ivermectin and albendazole, the former led to a greater rate of parasitological cure (RR 1.79, 95% CI 1.55 to 2.08), while having a safety profile that was comparable [59]. Moreover, in clinical trials that compared ivermectin with thiabendazole, there was little to no difference in parasitological cure (RR 1.07, 95% CI 0.96 to 1.20). On the other hand, ivermectin was associated with a lower risk of adverse events (RR 0.31, 95% CI 0.20 to 0.50).

In addition, in studies that compared various doses of ivermectin, administering a second dose of 200 g/kg of ivermectin did not result in a greater rate of complete recovery in a select set of patients (relative risk 1.02, 95% confidence interval [CI] 0.94 to 1.11; 94 participants, two trials) [59].

Moreover *Strongyloides*/CMV overlapping infections have been reported to be associated with bacterial superinfections, as shown in this review with concomitant bacterial infections in nine patients (42.8%). Five of these (23.8%) were caused by Gram-negative bacteria.

In order for the cycle of *Strongyloides* autoinfection to continue, filariform larvae must first break through the mucosa of the gastrointestinal tract, then enter the circulatory system, and finally reach the lungs, where they must break through the alveolar spaces [58]. When larvae penetrate the mucosa of the gastrointestinal tract, this frequently results in the translocation of enteric bacteria and bacteremia [58]. Link and colleagues examined 38 cases of severe bacterial infection that occurred in the context of hyperinfection. They discovered that 73% of the patients had bacteremia [60]. The onset of severe bacterial infections in conjunction with hyperinfection with *Strongyloides* poses a large mortality risk that can reach as high as 86% [60].

This narrative review presents different limitations. First, this is a narrative revision of the literature and lacks a systematic methodology or meta-analysis of the data. Secondly, cases reported are uncommon in the literature and the whole population is small with respect to other more frequent co-infections in CMV-infected patients reported in the literature (i.e., bacterial or fungal).

## 5. Conclusions

In this review, we have summarized the literature regarding CMV/*Strongyloides* infections in immunocompromised hosts. The main idea behind this work is to raise awareness of concomitant *Strongyloides* infections in immunosuppressed patients with isolated or disseminated CMV disease. In fact, CMV/*Strongyloides* co-infections are characterized by high morbidity and mortality, despite early diagnosis and treatment. Moreover, we presented the first European case of CMV/*Strongyloides* infections reported in the literature to our knowledge.

## Figures and Tables

**Table 1:** Manuscripts collected for the narrative review.

First Author et al. (Year)	Type of Study	Origin of Patient(s)
Rehman et al. (2007) [10]	Case Report	1, Oman
Elzein et al. (2016) [11]	Case Report	1, Saudi Arabia
Raj et al. (2020) [12]	Case Report	1, Indian
Soman et al. (2002) [13]	Case Report	1, India
Brugemann et al. (2010) [14]	Case Report	1, U.S.
Roseman et al. (2013) [15]	Case Report	1, Honduras (Donor)/U.S. (Recipient)
Steiner et al. (2002) [16]	Case Report	1, U.S.
Nordheim et al. (2018) [17]	Case Report	1 Donor NA; 2 Recipients Caucasian
Vilela et al. (2008) [18]	Case Report	1, Brazil
Fallahzadeh et al. (2021) [19]	Case Report	1, U.S.
Venizelos et al. (1980) [20]	Case Report	1, Puerto Rico
Narasimhan et al. (2021) [21]	Case Report	1, India
Hoy et al. (1981) [22]	Case Report	1, U.S.
Wang et al. (1999) [23]	Case Report	1, Puerto Rico
Crowe et al. (2019) [24]	Case Report	1, originally from Guyana and immigrated to the U.S. approximately 5 years before presentation
Rathor et al. (2016) [25]	Case Report	1, Indian
Rahman et al. (2020) [26]	Case Report	1, Jamaica
Al-Shyoukh et al. (2020) [27]	Case Report	1, U.S.
Ashida et al. (2020) [28]	Case Report	1, Japan
Weiser et al. (2011) [29]	Case Report	1, U.S.
Miglioli-Galvão et al. (2020) [30]	Case Series	10, Brazil

Abbreviations: NA: not available; U.S.: United States.

**Table 2:** Clinical and microbiological outcomes of patients with overlapping *Strongyloides*/CMV infections from the available literature.

N of patients			21
Male (N/%)			14 (77.8%)
Age (median, IQR, range)			47 (37–59) (20–68)
Underlying condition:			
	SOT (N/%)		12 (57.1%)
		Renal (N/%)	9 (75%)
		Liver (N/%)	1 (8.3%)
		Heart (N/%)	1 (8.3%)
		Pancreas (N/%)	1 (8.3%)
	Haematological diseases (N/%)		3 (14.2%)
	HIV/AIDS (N/%)		2 (9.5%)
	Reumatologic diseases (N/%)		3 (14.2%)
	None		1 (4.8%)
Steroids therapy (N/%)			16 (76.1%)
Other immunomodulatory treatment			13 (61.9%)
	Tacrolimus (N/%)		9 (42.8%)
	Azathioprine		3 (14.2%)
	MMF		8 (38.1%)
	Others		5 (23.8%)
	Multiple-drug therapy		8 (38.1%)
Comorbidities			6 (28.5%)
	Hypertension		4 (19.1%)
	Hypothyroidism		2 (9.5%)
	DM		2 (9.5%)
Symptoms			
GEL symptoms			16 (76.1%)
Skin symptoms			3 (14.2%)
Respiratory symptoms			6 (28.5%)
CMV manifestations:	Isolated reactivation		16 (76.1%)
	Disseminated disease		4 (19.1%)
	GEL localization		1 (4.8%)
*Strongyloides* manifestations:	HS		14 (66.7%)
	GEL localization		6 (28.5%)
	Hypereosinophilia		1 (4.8%)
Treatment	CMV	Ganciclovir	10 (47.6%)
		Valgaciclovir	2 (9.5%)
		None	9 (42.8%)
	*S. stercoralis*	Ivermectin	14 (66.7%)
		Thiabendazole	3 (14.2%)
		Albendazole	3 (14.2%)
		Mebendazole	1 (4.8%)
		Multiple therapy	3 (14.2%)
		None	1 (4.8%)
Concomitant bacterial infection			9 (42.8%)
	Gram − ve		5 (23.8%)
	Gram + ve		4 (19.1%)
Concomitant viral infection			1 (4.8%)
Concomitant fungal infection			1 (4.8%)
Concomitant protozoic infection			1 (4.8%)
Outcome	Alive		10 (47.6%)
	Death		11 (52.4%)

Abbreviations: N: number; IQR: interquartile range; SOT: solid organ transplant; HIV: human immunodeficiency virus; AIDS: acquired immunodeficiency syndrome; MMF: mycophenolate; DM: diabetes mellitus; CMV: Cytomegalovirus; HS: hyperinfection syndrome; GEL: gastroenterological; *S. stercoralis*: *Strongyloides stercoralis*; −ve: negative; +ve: positive.

## Data Availability

Not applicable.
